# Doxycycline Prophylaxis for Skin and Soft Tissue Infections in Naval Special Warfare Trainees, United States^1^

**DOI:** 10.3201/eid3001.230890

**Published:** 2024-01

**Authors:** Jeffrey Spiro, Piotr Wisniewski, Julia Schwartz, Alfred G. Smith, Sara Burger, Drake H. Tilley, Ryan C. Maves

**Affiliations:** Naval Medical Center, San Diego, California, USA (J. Spiro, P. Wisniewski, D.H. Tilley, R.C. Maves);; Naval Health Research Center, San Diego (P. Wisniewski);; Naval Special Warfare Center, Coronado, California, USA (J. Schwartz, S. Burger);; Naval Medical Center, Portsmouth, Virginia, USA (A.G. Smith);; Wake Forest University School of Medicine, Winston-Salem, North Carolina, USA (R.C. Maves)

**Keywords:** bacteria, doxycycline, prophylaxis, skin and soft tissue infection, SSTI, saltwater exposure, military, United States

## Abstract

In 2015, several severe cases of skin and soft tissue infection (SSTI) among US Naval Special Warfare trainees prompted the introduction of doxycycline prophylaxis during the highest-risk portion of training, Hell Week. We performed a retrospective analysis of the effect of this intervention on SSTI incidence and resulting hospital admissions during 2013–2020. In total, 3,371 trainees underwent Hell Week training during the study period; 284 SSTIs were diagnosed overall, 29 of which led to hospitalization. After doxycycline prophylaxis was introduced, admission rates for SSTI decreased from 1.37 to 0.64 admissions/100 trainees (p = 0.036). Overall SSTI rates remained stable at 7.42 to 8.86 SSTIs/100 trainees (p = 0.185). Hospitalization rates per diagnosed SSTI decreased from 18.4% to 7.2% (p = 0.009). Average length of hospitalization decreased from 9.01 days to 4.33 days (p = 0.034). Doxycycline prophylaxis was associated with decreased frequency and severity of hospitalization for SSTIs among this population.

Skin and soft tissue infections (SSTIs) are common and potentially severe medical conditions that occur regularly in both military and nonmilitary populations. Military members, however, have a 21% higher incidence of SSTI than do similarly aged nonmilitary populations because of a variety of factors, such as increased rate of minor traumatic injuries and intermittently poor hygiene practices often associated with field exercises and deployments to austere locations (*1*). Furthermore, a disproportionate burden of SSTI in the military occurs in recruit and training settings, likely resulting from increased crowding, decreased adherence to good personal hygiene, and environmental contamination (*1*,*2*).

United States Naval Special Warfare (NSW) training in Coronado, California, USA, exposes trainees to extreme physical stresses and environmental conditions that create an ideal setting for increased frequency and severity of SSTIs. In 2015, medical staff at the Naval Special Warfare Center (NSWCEN) identified a concerning trend in its trainees, when a series of severe SSTIs were identified as being caused by invasive gram-negative saltwater-associated pathogens, including *Shewanella algae* and *Vibrio* spp. (*3*). Those severe SSTIs primarily occurred during the highest-risk phase of training, Hell Week, which consists of 5 days of intense exertion with minimal rest and continuous exposure to wet and cold environments.

As a result, starting in September 2015, all NSW trainees entering Hell Week received 100 mg oral doxycycline daily as prophylaxis primarily aimed at limiting the incidence of severe SSTIs, particularly those caused by invasive gram-negative saltwater-associated pathogens. Trainees started prophylaxis 2 days before Hell Week and stopped the day after completing the training exercise (8 total doses). For this study, we reviewed data from 2013–2020 to determine whether this intervention had any significant effect on the rate, severity, or quality of SSTIs in the NSW trainee population during Hell Week.

## Methods

Our study was a retrospective cohort study of all NSW trainees who participated in the Hell Week phase of training during April 2013–February 2020 at the NSW Center in Coronado, California. We selected this timeframe because of limited data availability: NSWCEN used nonelectronic medical records before April 2013 that were no longer available at the time of this analysis. The study was approved as minimal risk with a waiver of informed consent by the Naval Medical Center San Diego (NMCSD) institutional review board on July 26, 2021 (approval no. NMCSD.2021.0007). 

Overall, 42 separate Hell Weeks occurred during the study timeframe, roughly every 2 months. NMCSD is the principal care location for NSW trainees requiring emergency or inpatient care. To this end, we requested hospital admission data for all NSW trainees admitted to NMCSD during the study period. We narrowed the search further by only requesting data from the 42 Hell Week periods. Each Hell Week period was specifically defined as the training exercise itself (5 days) plus the 7 days after its completion. This timeframe helped to account for SSTIs that likely began during Hell Week but were not diagnosed until several days after its conclusion. Once admission data were obtained, investigators (primarily J. Spiro) reviewed data from each admission to determine whether the primary reason for hospitalization was treatment of an SSTI. Once those target cases were identified, we compiled and analyzed relevant data points. Separately, a limited amount of deidentified data was also provided from the NSWCEN medical staff directly. Those data consisted only of the number of NSW trainees who participated in each Hell Week, as well as the number of SSTIs diagnosed locally at the NSWCEN clinic during each Hell Week period, regardless of whether that trainee ultimately required hospitalization. The case definition of SSTI at NSWCEN was consistent throughout the study period and was based on clinical manifestations, mainly skin erythema, edema, and warmth with or without concurrent abscess, which led to initiation of treatment-dose antimicrobial therapy. Of note, data regarding common side effects related to doxycycline use were not readily available from NSWCEN, so we did not analyze side effect incidence during the study timeframe. Although specific documentation of medication adherence was not available, all doxycycline doses were administered under directly observed therapy by NSWCEN medical personnel for the duration of prophylaxis. Last, to assess for development of resistance to doxycycline over the study timeframe, the NMCSD microbiology department provided antibiotic susceptibility data for blood and wound cultures positive with *Staphylococcus aureus* from all NSW trainees during the study period, regardless of whether they were participating in Hell Week or hospitalized for an SSTI.

The primary outcome of this study was hospital admission rates for SSTI during Hell Week periods before and after initiating doxycycline prophylaxis. September 2015 was the first Hell Week period during which trainees received doxycycline prophylaxis and marked the beginning of the post-doxycycline prophylaxis cohort. Secondary outcomes included overall incidence of SSTIs (regardless of hospital admission), percentage of diagnosed SSTIs that led to hospitalization, length of hospitalization, and assessment for notable changes in blood and wound culture data before and after the doxycycline prophylaxis. The NMCSD microbiology department performed all blood and wound culture analysis per hospital protocol and procedures did not differ during the study period. Minimal changes were made to training protocols during Hell Week at NSWCEN during the study timeframe, so the preintervention cohort served as the functional control group for this analysis. In addition, given that Hell Week periods occurred roughly every 2 months during the study timeframe, their seasonal distribution did not meaningfully differ before and after doxycycline prophylaxis, and we did not perform analysis incorporating seasonal incidence of disease.

Statistical analysis of hospital admission rates for SSTI, general incidence of SSTI, and percentage of diagnosed SSTI leading to hospitalization all involved comparison of incidence rates for which we determined 2-tailed z-scores with a p value cutoff of <0.05. We used the Byar method to develop rate ratios when applicable. We treated length of hospital stay before and after the intervention as independent groups and analyzed them with unpaired t-tests, again using a p value of <0.05 as significant. We used OpenEpi version 3 (https://www.openepi.com/Menu/OE_Menu.htm) for calculations. We limited analysis of blood and wound culture data to descriptive statistics given the more nuanced nature of the data and the smaller sample size.

## Results

A total of 3,371 NSW trainees participated in the 42 Hell Week training periods during April 2013–February 2020. From that population, 70 patients were admitted to NMCSD for all medical conditions; 29 of those admissions were primarily for treatment of SSTI. Specific demographic data on the 3,371 trainees were limited because of variable attrition rates in each class throughout the training process. However, all trainees were men 18–33 years of age. Most trainees were enlisted personnel, and most enlisted and officer trainees had no previous active military experience. The 29 trainees hospitalized for SSTIs were all men; age range was 19–28 years (mean 23.4 years). Of 29 hospital admissions, 13/29 (44.8%) occurred during Hell Week, and 16/29 (55.2%) occurred in the 7 days after each Hell Week. Mean time from start of Hell Week to hospital admission was 6.24 days. Twenty cases involved SSTI in 1 or both lower extremities, 3 cases involved a combination of upper and lower extremities, and 6 cases involved an isolated upper extremity. Seven patients had clear abscesses or another complication that required incision and drainage or other surgical intervention. Four patients required treatment in the intensive care unit. No deaths occurred among the 29 trainees hospitalized for SSTI during the study period.

Doxycycline prophylaxis during Hell Week started in September 2015. Fifteen Hell Weeks involving 1,024 trainees occurred before doxycycline prophylaxis began, and 27 Hell Weeks involving 2,347 trainees took place after the intervention. Fourteen of 29 cases of SSTI requiring hospital admission occurred before doxycycline prophylaxis was implemented, a rate of 1.37 hospitalizations/100 trainees. Fifteen of 29 cases took place after the intervention began, a rate of 0.64 hospitalizations/100 trainees. Those values correspond to a rate ratio of 0.468 (95% CI 0.226–0.968; p = 0.036) for SSTI leading to hospitalization after implementing doxycycline prophylaxis and a theoretical number needed to treat of 137.3 trainees to prevent 1 hospitalization for SSTI (Table 1).

**Table 1:** Primary and secondary outcome data in study of doxycycline prophylaxis for SSTIs in Naval Special Warfare trainees, United States*

Outcome	Preprophylaxis	Postprophylaxis	Rate ratio (95% CI)	p value
Hospital admission rate per 100 trainees	1.37 (14/1,024)	0.64 (15/2,347)	0.468 (0.226–0.968)	0.036
Mean hospital admission length, d	9.07	4.33	NA	0.034
Overall SSTI incidence rate per 100 trainees	7.42 (76/1,024)	8.86 (208/2,347)	1.19 (0.918– 1.55)	0.185
Hospital admission rate per diagnosed SSTI	18.4 (14/76)	7.2 (15/208)	0.392 (0.189–0.811)	0.0089

*Values are % (no. patients/total no. in category) except as indicated. NA, not applicable; SSTI, skin and soft tissue infection.

A total of 284 SSTIs were diagnosed locally at the NSWCEN medical clinic during the study period; 76 infections occurred before the doxycycline prophylaxis was enacted and 208 after (Figure). The more general incidence of SSTI before the intervention was 7.42 SSTIs per 100 trainees, whereas the incidence after the intervention was 8.86 SSTIs per 100 trainees. Those data correspond to a rate ratio of 1.19 (95% CI 0.918–1.55; p = 0.185) for SSTI after doxycycline prophylaxis was begun. In the preintervention period, 14 hospitalizations among 76 SSTI cases resulted in an 18.4% admission rate, whereas 15 hospitalizations among 208 SSTIs in the postintervention period produced a 7.2% admission rate. The hospitalizations per infection rate ratio was 0.392 (95% CI 0.189–0.811; p = 0.0089) after doxycycline prophylaxis was started (Table 1). The average length of hospitalization in trainees admitted for SSTI in the predoxycycline prophylaxis period was a mean of 9.07 days (median 7 days); average length in the postdoxycycline prophylaxis period was a mean of 4.33 days (median 4 days). The difference of those means was 4.74 days (95% CI 0.40–9.08; p = 0.034) (Table 1).

**Figure:**
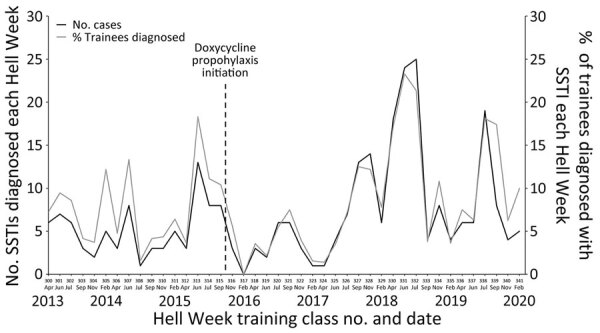
Total number of SSTIs diagnosed at the Naval Special Warfare Center medical clinic during each Hell Week with corresponding percentage of Naval Special Warfare trainees diagnosed with SSTI in each training class, United States. Each Hell Week is labeled by month, year, and training class number (300–341). Date of doxycycline prophylaxis initiation is indicated. SSTI, skin and soft tissue infection.

Blood and wound culture data from the 29 trainees hospitalized for SSTI revealed 7 case-patients with positive wound cultures and 3 cases of bacteremia before initiation of doxycycline prophylaxis. In the postintervention population, data revealed 8 case-patients with positive blood cultures and 2 cases of bacteremia. *S. aureus* and *Streptococcus pyogenes* were the most commonly identified pathogens both before and after doxycycline prophylaxis was introduced (Table 2). We observed a notable change in invasive infections with gram-negative saltwater-associated pathogens. Four cases of *S. algae* infection (3 from wound culture, 1 from blood culture) and 2 cases of *Vibrio* spp. (from both wound and blood cultures) were identified preintervention, whereas 1 case of *S. algae* infection (from blood culture) and 1 case of *Vibrio* spp. infection (wound culture) were seen postintervention.

**Table 2:** Wound and blood culture data from Naval Special Warfare trainees hospitalized for skin and soft tissue infections in the periods before and after implementation of doxycycline prophylaxis, United States

Pathogen	Preprophylaxis		Postprophylaxis	
	Positive wound cultures	Positive blood cultures	Positive wound cultures	Positive blood cultures
*Staphylococcus aureus*	6	1	5	1
Group A *Streptococcus*	4	0	4	0
*Shewanella algae*	3	1	0	1
*Vibrio spp.*	2	1	1	0
Coagulase-negative *Staphylococcus*	2	0	1	1
*Staphylococcus lugdunensis*	1	0	0	0
*Diptheroids*	1	0	2	0
*Enterococcus faecalis*	2	0	0	1
*Acinetobacter hemolyticus*	2	0	0	0
*Pseudomonas aeruginosa*	1	0	0	0
*Providencia rettgeri*	0	0	1	0

Blood and wound culture data regarding *S. aureus* antibiotic susceptibility in all NSW trainees throughout the study period (including those outside of Hell Week training) showed 37 *S. aureus* isolates with documented tetracycline class sensitivities, reported for minocycline in all cases. There were 10 isolates in the predoxycycline cohort, all of which were sensitive to minocycline (MIC <0.5). In the postdoxycycline cohort, 27 isolates had documented sensitivities; 25 were minocycline-sensitive (MIC <0.5), 1 had intermediate sensitivity to minocycline (MIC <8), and 1 was resistant to minocycline (MIC >16).

## Discussion

The principal finding of our study was that the introduction of doxycycline prophylaxis to NSW trainees entering Hell Week was associated with a decreased incidence of hospitalization for severe SSTI. In addition, secondary findings of significantly decreased rate of hospitalization per diagnosed SSTI and decreased length of hospitalization after doxycycline prophylaxis further support an overall decrease in the severity of SSTIs in the study population. The overall incidence of SSTI (regardless of hospitalization status) did not change significantly, possibly suggesting that mild-to-moderate SSTIs were less affected by doxycycline prophylaxis. This application of doxycycline prophylaxis focused on reducing severe SSTIs caused by occupational or recreational exposure. Other investigations into doxycycline as prophylaxis against skin infection have mostly centered on prevention of iatrogenic complications, such as surgical site infections (*4*–*6*), or severe infections related to toxicities of antineoplastic agents, such as cetuximab or panitumumab (*7*,*8*). In military populations, efforts to prevent SSTIs (largely aimed at *S. aureus* infections) have been limited to decolonizing strategies, such as intranasal mupirocin or topical chlorhexidine, in addition to several more recent vaccination trials (*9*).

Doxycycline has a well-established history for use as prophylaxis against other infections, such as malaria (*10*,*11*) and, more recently, syphilis (*12*–*14*). Doxycycline has also been used as prophylaxis against potential bioterrorism agents, such as anthrax and tularemia (*15*,*16*), as well as against Lyme disease (*17*), leptospirosis (*18*), and other zoonoses. Dosing of doxycycline for those purposes has varied depending on the study, but typically is either intermittent 200 mg doses (given either before or after exposure) or daily 100 mg doses. Pharmacokinetic data indicate that a 200 mg dose is largely eliminated from the body after roughly 3–5 days, and some studies have questioned the efficacy of 200 mg weekly dosing schedules (*19*,*20*). Regardless, the choice of dosing schedule is typically tailored to the recipient and environment, particularly because less frequent drug administration might sometimes be the only practical option. Overall, this large body of evidence is indicative of doxycycline’s relative safety and tolerability for populations at high risk for polypharmacy or drug side effects.

The nature and intensity of NSW training places the subjects of this study at particularly high risk for SSTIs, both from common causes like *S. pyogenes* and *S. aureus* and from rarer saltwater-associated organisms, such as *Vibrio* spp. and *S. algae*. The reasons for this higher risk are multifactorial. NSW trainees during Hell Week experience substantial and often diffuse skin breakdown and extremity edema because of the intensity of the physical training and near-constant exposure to ocean water. In addition, NSW trainees might have a component of functional immune impairment as a result of minimal sleep, high stress, and frequent transmission of more minor communicable illnesses. Antibiotic prophylaxis in such settings is not novel; chemoprophylaxis has long been used to target invasive infections from group A *Streptococcus* in military training populations (*21*). The doxycycline prophylaxis analyzed in this study was specifically started in 2015 to limit severe invasive infections caused by saltwater-associated gram-negative pathogens such as *Vibrio* spp. and *S. algae* in the setting of a previous outbreak. Doxycycline is still considered a first-line agent for treating *Vibrio* spp. SSTIs with minimal documented resistance (*22*,*23*). Invasive *S. algae* infections are uncommon in otherwise young and healthy persons, as discussed in a recent case series from our center (*3*). Antibiotic susceptibility patterns are less well studied, but data from some areas show high rates of susceptibility to third-generation cephalosporins, fluoroquinolones, tetracycline, and aminoglycosides (*24*).

A review of doxycycline as long-term malaria prophylaxis by the Centers for Disease Control and Prevention in 2011 found mild-to-moderate gastrointestinal symptoms (nausea, abdominal pain) to be the most common adverse effect, occurring in 4%–33% of patients (*25*). An investigation of US Marines in Okinawa, Japan, in 2014 taking 200 mg doxycycline weekly reported side effects, primarily nausea, in 18% of 291 study participants. Peace Corps volunteers receiving long-term malarial prophylaxis reported gastrointestinal side effects in 40% of survey respondents taking doxycycline, but few of those persons reported severe symptoms, and symptoms did not frequently lead participants to discontinue the medication (*26*). More severe gastrointestinal manifestations, such as pill-induced esophagitis, occur in <1% of doxycycline recipients, although that number might be an underestimate (*25*,*27*,*28*). Phototoxicity has been reported in 7%–21% of persons taking doxycycline for long-term malaria prophylaxis, depending on the degree of sun exposure (*25*,*29*). Most of those side effects might be mitigated through some simple precautions (e.g., remaining upright after taking medications and taking them with adequate water to prevent pill esophagitis, wearing sunscreen and avoidance of direct skin exposure to sun for phototoxicity). In our investigation, a previously healthy population was exposed to doses of 100 mg doxycycline daily for 8 days. Although specific side effects were not actively solicited at the time, we noted no overt increase in attributable symptoms.

One possible concern with the type of intervention analyzed in this study is development of resistance against doxycycline. One relevant retrospective study from 2016 (*30*) analyzed 168 *S. aureus* isolates collected from wounded US military personnel returning from Iraq and Afghanistan during 2009–2012 with regard to tetracycline class resistance and doxycycline exposure, primarily in the form of long-term antimalarial prophylaxis. That study found overall resistance rates of 23% (tetracycline), 15% (doxycycline), and 14% (minocycline) and identified no significant association between doxycycline exposure and resistance to the tetracycline antimicrobial class (*30*). In our study, we assessed *S. aureus* sensitivity to tetracycline class antibiotics from blood and wound cultures and found 2 of 27 isolates with poor sensitivity (1 with intermediate resistance and 1 fully resistant) in the postdoxycycline prophylaxis period, compared with no documented resistance in the predoxycycline period. However, the 2 nonsensitive isolates were from 2015 and 2016 (relatively recently after initiation of doxycycline prophylaxis), and no documented resistance was seen in the 22 isolates from 2017–2020. On the basis of those limited data, we did not identify a concerning pattern for development of resistance to tetracycline-class antibiotics over the course of the study period. In addition, the intervention we analyzed was periodic, rather than continuous, possibly reducing any selective pressure to promote widespread resistance. Furthermore, the otherwise healthy population of trainees is unlikely to have extensive interaction within the medical setting after their Hell Week training, thereby reducing the likelihood of spreading more resistant clones to potentially immune-suppressed patients.

The first limitation of our study is that it was not a randomized trial, where rates of infection can be directly compared with minimal bias. However, given the limited changes to Hell Week training over the past 10 years, as well as the generally stable demographics of NSW trainees, the study population before doxycycline prophylaxis can reasonably be considered internal controls in this setting. Second, this study was a retrospective analysis, preventing the assignment of any true causality to doxycycline prophylaxis. Data regarding adverse reactions in the study population were not reliably obtainable, and therefore we cannot be certain that a rise in minor iatrogenic issues was not present. In addition, after doxycycline prophylaxis was implemented, NSWCEN medical providers’ increased awareness of serious SSTI complications could have led to their recognizing and treating infections earlier, ultimately limiting the severity of illness in the postintervention cohort. Finally, the total number of hospital admissions for SSTI was relatively small, thereby increasing the chance that the lower incidence of SSTI noted after the intervention was coincidental.

The overall goal of this study was to determine whether introducing doxycycline prophylaxis to NSW trainees entering Hell Week had an effect on the severity, rate, and quality of SSTI. Our data indicate that doxycycline prophylaxis in this population was associated with a significantly decreased rate of hospitalization for SSTI, decreased rate of hospitalization per identified SSTI, and a decreased length of hospitalization for SSTI treatment. General rates of SSTI diagnosed by clinic medical providers were not significantly different before and after doxycycline prophylaxis. Fewer cases of severe SSTI caused by invasive gram-negative saltwater-associated pathogens (*Shewanella* spp., *Vibrio* spp.) were associated doxycycline prophylaxis, but because of the small number of those infections, we did not measure the decrease statistically.

In conclusion, doxycycline appears generally well tolerated and was associated with a decrease in severe SSTIs requiring hospitalization, as well as a decrease in cases caused by aquatic gram-negative pathogens. In special populations whose circumstances include prolonged saltwater exposure and participation in activities that place them at high risk for skin trauma, doxycycline prophylaxis at the time of highest risk appears safe and might reduce the risk for severe SSTIs.
